# Salt Marsh Elevation Drives Root Microbial Composition of the Native Invasive Grass *Elytrigia atherica*

**DOI:** 10.3390/microorganisms8101619

**Published:** 2020-10-21

**Authors:** Edisa García Hernández, Elena Baraza, Christian Smit, Matty P. Berg, Joana Falcão Salles

**Affiliations:** 1 Microbial Community Ecology, Groningen Institute for Evolutionary Life Sciences, University of Groningen, 9747 AG Groningen, The Netherlands; 2 Departamento de Biologia, Universitat de les Illes Balears-INAGEA, 07122 Mallorca, Spain; elena.baraza@uib.es; 3 Community and Conservation Ecology Group, Groningen Institute for Evolutionary Life Sciences, University of Groningen, 9747 AG Groningen, The Netherlands; c.smit@rug.nl (C.S.); m.p.berg@vu.nl (M.P.B.); 4 Department of Ecological Science, Section Animal Ecology, Vrije Universiteit Amsterdam, 1081 HV Amsterdam, The Netherlands

**Keywords:** *Elytrigia atherica*, salt marsh, elevation, root bacterial communities

## Abstract

*Elytrigia atherica* is a native invasive plant species whose expansion on salt marshes is attributed to genotypic and phenotypic adaptations to non-ideal environmental conditions, forming two ecotypes. It is unknown how *E. atherica*–microbiome interactions are contributing to its adaptation. Here we investigated the effect of sea-water flooding frequency and associated soil (a)biotic conditions on plant traits and root-associated microbial community composition and potential functions of two *E. atherica* ecotypes. We observed higher endomycorrhizal colonization in high-elevation ecotypes (HE, low inundation frequency), whereas low-elevation ecotypes (LE, high inundation frequency) had higher specific leaf area. Similarly, rhizosphere and endosphere bacterial communities grouped according to ecotypes. Soil ammonium content and elevation explained rhizosphere bacterial composition. Around 60% the endosphere amplicon sequence variants (ASVs) were also found in soil and around 30% of the ASVs were ecotype-specific. The endosphere of HE-ecotype harbored more unique sequences than the LE-ecotype, the latter being abundant in halophylic bacterial species. The composition of the endosphere may explain salinity and drought tolerance in relation to the local environmental needs of each ecotype. Overall, these results suggest that *E. atherica* is flexible in its association with soil bacteria and ecotype-specific dissimilar, which may enhance its competitive strength in salt marshes.

## 1. Introduction

Plants may show phenotypic and genotypic adaptations to particular stressful environmental conditions. Plant adaptation can be modulated by their associated microbes, which can contribute to fitness by providing metabolic capabilities and by modulating pathways’ increasing tolerance of plants to abiotic and biotic stresses or nutrient-limiting conditions [1,2]. Some of these phenotypic changes are expressed in response to an environmental cue, for example mycorrhizal fungi can change root morphology in low-P soils [3,4] allowing plants to successfully inhabit previously non-ideal environments [5,6,7,8]. This example highlights the fact that plant-microbe interactions can provide fitness benefits to the host, and therefore are expected to be under selection [5]. Although the ecological aspects of plant genotype and microbiome interactions have been largely studied, especially in the context of agriculture, the consequences of this microbially driven phenotypic plasticity on plant evolution remains poorly understood [9,10,11]. This evolutionary perspective, which requires an overview of patterns in plant–microbiome interactions and phenotype responses in natural ecosystems, might play an important role in plant range expansion and adaptation to global change scenarios. In this context, placing invasive plant species under the perspective of holobiont (plants plus associated microbiome) [12] might improve our understanding of the mechanisms driving successful adaption and competitive advantage in new environmental conditions [13].

In salt marsh ecosystems, plants are distributed along the elevational gradient according to their sea-water inundation tolerance. Plants with ecophysiological traits that promote resistance to higher flooding frequency and inundation time, such as aerenchyma formation, conservative water use, or the production of N-rich solutes for osmotic adjustment [14,15], usually occur at low elevations (LE) [16,17,18], whereas plants lacking these traits have a higher fitness at high elevations (HE). Sea coach *Elytrigia atherica* is an example of the latter. It is a dominant species in the late stage of the salt marsh natural succession in areas that are usually located higher above sea level due to silt accretion [19,20]. This tall grass stands out for being highly competitive, as demonstrated by its wide distribution—the species occurs in salt marshes across the North Atlantic Coast from Northern Portugal to Southern Denmark—and for reducing the plant diversity of natural salt marsh communities at high elevation sites [21]. *Elytrigia atherica* is expanding into recently drained low elevation and younger sites, where it outcompetes most other mid-successional species, causing a reduction in plant diversity that is comparable to an exotic species invasion [21,22]. Its successful expansion to the low marsh with altered (a)biotic conditions has been attributed to the high chance of establishment once a seedling has germinated, due to clonal spread, its ability to form aerenchyma and the relative unpalatability for salt marsh grazers, such as geese [16,23,24,25]. The different conditions found in low elevation salt marsh compared to high elevation marshes have led to the formation of two *E. atherica* ecotypes with a genetic differentiation in plants that grow at a distance longer than 100 m apart [23] but also within patches of <5 m [22]. Furthermore, phenotypic differences have been observed in traits such as shoot length, number of spikelets per spike and ramet numbers between HE and LE ecotypes [23]. One factor that potentially could also impact the invasiveness of this plant species is the extent to which the LE and HE ecotype of *E. atherica* differ in relation to their association with beneficial microbes, which could contribute to their extension into LE salt marsh sites.

Plant–microbe associations have an important role in plant competitive ability in terms of establishment, invasion and persistence in habitats. For example, comparing invasive species against rare plants, the former have shown a higher positive soil feedback attributed to a slower accumulation of pathogens compared to the latter [26]. Furthermore, some endophytes that drive plant dominance are able to inhabit seeds, which represents an advantage for its transmission to new generations and introduction to new habitats [27]. Moreover, the benefits of the association with plant promoting bacteria also provides advantages in some plant invasive species compared to native species [28]. Altogether, these examples highlight the importance of microbes in invasion processes. However, the role of microbial interactions in plant invasion success in multi-stressed ecosystems, such as salt marshes, is currently poorly known [29]. 

The aim of the present study was to assess the extent to which the association between *E. atherica* and its bacterial communities was affected by elevation and its associated soil biotic (mycorrhiza presence, soil bacterial communities) and abiotic factors (flooding frequency, pH, nutrient content). Differences in plant-associated bacteria between salt marsh elevations could indicate that bacteria can potentially play an important role in *E. atherica*’s range expansion within sites across elevations. We therefore sampled plants as well as rhizosphere and bulk soils from LE and HE sites in three locations across a salt marsh of a barrier island. Given that the level of interaction between plant roots and associated microbiome might be dependent on microbiome localization, we sampled the free-living soil bacterial communities, as well as those living around (rhizosphere) or in the roots (endosphere). Additionally, potential factors influencing these interactions, such as plant traits, root mycorrhizal colonization and soil physicochemical parameters were measured. We hypothesized that (i) salt marsh elevation will lead to differences in soil properties with higher levels of soil moisture and sodium content at LE and higher mycorrhizal colonization in HE plants. Moreover, we tested whether (ii) elevation drives soil, rhizosphere and endosphere bacterial (functional) composition. We expected that soil and rhizosphere bacterial composition, and their potential functions, are driven by elevation and thus inundation frequency. Moreover, we expected that the compositions of endosphere bacterial communities are partially independent from rhizosphere and bulk soil communities and specific to each plant ecotype, and therefore vary with the salt marsh elevation.

## 2. Materials and Methods

### 2.1. Study Sites and Sampling Strategy

Plant and soil samples were collected from the salt marsh of the barrier island of Schiermonnikoog (53°29′ N, 6°10′ E), the Netherlands in July 2017. This island presents a well-documented salt-marsh chronosequence [19]. In salt marshes, elevation determines the frequency, amplitude, and duration of sea-water inundation events [24]. We selected sites with high plant coverage (>90%) where *E. atherica* was dominant. Three sites at high elevation (HE) (>1.6 m Amsterdam Ordnance Data, AOD) and three sites at low elevation (LE) (<1.400 m AOD) (Table 1). Site elevation was measured using a real-time kinetimatic differential GPS (RTK-dGPS, Leica Viva GS12 GNSS receiver and CS15 controller), with a vertical accuracy of less than 2 mm. HE sites were indicated as H1–H3 and LE sites as L1–L3 (Supplementary Material S1). Inundation frequency was expressed as the amount of times per year a site was flooded and was calculated using a model, which was based on the seawater-level fluctuation in relation to the natural elevation of each site [30]. At each site, three sampling plots of approximately 5 × 5 m were selected. Salt-marsh age of each sampling site was estimated based on [30], HE sites were estimated older than LE sites, except L1, which has the same age as H1 (Table 1). Subdominant plant species composition varied at each sampling site (Table 1). QGis version 3.8 was used to make the map in Supplementary Material S1.

### 2.2. Plant and Soil Sampling

In each plot, three PVC cylinders (Ø 10 cm; 10 cm high) were hammered into the soil, dug out with the alive *E. atherica* plants and placed in sterile plastic bags. For measuring the Specific Leaf Area (SLA), the third leave counting from the top of three healthy plants from each plot were clipped and pasted on a sheet of paper with transparent tape. The sheets contained scales of known length for calibration and were scanned. Leaf area was calculated using ImageJ version 1.52n (US National Institutes of Health). Leaves were dried in the oven at 60 °C for 72 h, weighted (to the nearest 0.1 mg) and SLA was calculated by dividing the sum of the three leaves’ area by the total dry mass [31].

In each plot we measured the plant biomass by clipping and collecting all above ground tissue in a 20 × 20 cm squares. Plant litter was collected in the same squares by hand and placed in a paper bag. At the laboratory, the stem and leaves of *E. atherica* were separated from other plant species and dried at 70 °C for 48 h and weighted. To quantify plant height in each plot we selected 10 reproductive, healthy-looking individuals and measured height from the beginning of the shoot until the end of the inflorescence [31]. The complete dataset of the plant traits, intensity of mycorrhizal colonization and plant litter biomass from each of the sampling sites are in Supplementary Material S2.

Six cores of bulk soil (Ø 3.5 cm: 10 cm depth) at random points inside the plot were taken and placed in a sterile plastic bag, which was sealed and transported to the laboratory on the same day. In the laboratory, any plant material was removed from the soil, after which the soil was sieved (4 mm mesh size) and homogenized to represent a composite sample. From each composite sample, 10 g of soil was placed in a sterile tube and frozen at −20 °C for DNA extraction. Approximately 500 g soil was kept at 4° C for physicochemical measurements and potential denitrification rate (PDR). Soil physicochemical parameters measured were texture, pH, moisture content, soil organic matter content (SOM), the content of sodium (Na), total carbon (TC), total nitrogen (TN), and nitrogen in nitrate and nitrite (N-NO^3−^, N-NO^2−^) and ammonium (N-NH^4+^). For detailed methods see Supplementary Material S3 and dataset in Supplementary Material S4.

Data analyses were carried out in R v3.6.2 (R-Core-Team, 2017). The collinearity between salt marsh height above sea level and estimated flooding frequency was VIF >10; therefore, we only report flooding frequency as a predictor variable. To test relationships between soil abiotic parameters, plant traits and site characteristics, a Principal Component Analysis (PCA) was applied to the scaled and centered variables using the “prcomp” function from the stats package.

### 2.3. Mycorrhizal Root Colonization

The percentage of root length infected with mycorrhiza was estimated by visual observation using a light microscope with 40 times magnification. Washed roots were cleared in 10% KOH and stained with 0.05% trypan blue in lactic acid (*v*/*v*), according to [32]. Root length infected by AM fungi was assessed using the magnified intersections method [33], where the frequency of colonization represents the ratio between the fragments of infected root and the total number of root fragments examined.

### 2.4. Rhizosphere and Endosphere Sample Preparation

Rhizosphere and endosphere DNA extraction was performed following standard procedures with some modifications [34]. Briefly, belowground plant tissue was separated from aboveground tissue using sterile scissors. Loose soil was manually removed, and the total root biomass was split for rhizosphere and endosphere procedures. The rhizosphere samples consisted of 4–5 g of roots and ~2 g rhizomes, placed in a flask containing 180 mL sterile sodium pyrophosphate (Na_4_PO_2_O_7_) (0.1%) to which some sterile 3-mm glass beads were added, shaken at 200 rpm at 25 °C for 1 h. The suspension was transferred to a 50 mL tube and 3200× *g* was centrifuged for 15 min. The resulting pellet containing the rhizosphere soil was stored at −20 °C for DNA extraction. The roots of endosphere samples were washed thoroughly with tap water until soil particles were not visible. Then, 4 g of roots and ~2 g rhizomes were placed into a flask containing 150 mL sterile distilled water plus 100 mL sterile 2% Tween 20 and sonicated for 5 min (Branson 1510 Ultrasonic Cleaner, Danbury, CT). The roots were then surface disinfected by immersion in 1.5% sodium hypochlorite and mixed at 200 rpm at 25 °C for 10 min, in 96% Ethanol (1 min) and sterile distilled water (3 times for 3 min each). To check for sterility, we took the last rinsing 100 µl of water and tissue from the last rinsing for blotting on R2A and 869 1/10 plates. The plates were checked for 2 to 5 days [35]. Samples without bacterial growth were considered surface sterilized and endophyte bacterial cells were extracted from the surface sterilized roots following [34]. Bacterial pellets were resuspended in 1.5% NaCl solution and stored at −20 °C for DNA extraction.

### 2.5. DNA Extraction and 16S rRNA Gene Sequencing

DNA from soil, rhizosphere and endosphere samples was isolated using 0.5 g of bulk soil or rhizosphere soil or 0.5 mL suspension, using the DNeasy Power Soil kit (QIAGEN) according to the manufacturer’s instructions. Except we performed the initial incubation with 50 µL lysozyme (10 mg mL^−1^) at 35 °C for 30 min and added 0.2 g 0.1 mm sterile glass beads to improve DNA isolation. Extracted DNA was quantified using the Nanodrop spectrophotometer (Thermo Scientific).

To partially amplify the 16S rRNA gene, 25 µL PCR reactions were performed in triplicate using the FastStart High Fidelity (Roche) kit following the protocol by [34] but using 10 ng of DNA sample instead of 5 ng. We used a 515F–926R primer set, spanning the variable region V4-5 [36]. The forward primer also contained a barcode sequence (10-mer) to allow pooling of multiple samples in one sequencing run. Amplicon size was confirmed in 1% agarose gels, and the three PCR products of each sample were pooled together to reduce PCR bias. PCR products were purified using the QIAquick PCR Purification Kit (Qiagen). The fluorescence of the purified amplicons was quantified using the Quant-iT PicoGreen ds DNA assay kit (Invitrogen, Carlsbad, CA, USA) on a TECAN infinite M200 Pro (Maennedorf, Switzerland) plate reader using at 485 nm excitation and 535 nm emission. Amplicons from all samples were pooled in equimolar concentration (30 ng/sample) and sequenced at Genewiz (South Plainfield, USA) on an Illumina MiSeq sequencer using a 2 × 300-bp read configuration. The obtained sequences were deposited in the database of the National Center for Biotechnology Information under the BioProject ID PRJNA642700.

### 2.6. Sequence Data Analysis

#### 2.6.1. Diversity Analyses

To join the pair-end sequences we used the Quantitative Insights into Microbial Ecology (QIIME) version 1.91 [37]. Demultiplexing and removing of primers were performed using the sequencing toolkit *cutadapt* [38]. Demultiplexed sequences were then imported into QIIME2 version 2018.2, and were quality filtered using the *deblur* algorithm [39] following the default parameters [40] except that the amplicons were trimmed to 381 bp length. A tree for phylogenetic diversity analyses was performed using the plugin *mafft*. Taxonomic identity to the Amplicon Sequence Variants (further on ASVs) was assigned using the classifier SILVA (version 132-2018) trained for the 515F/926R region with default similarity threshold of 0.7. The resultant feature table, taxonomy table and phylogenetic tree were then imported to R environment (R 3.6, R-Core-Team, 2017).

Chloroplasts, mitochondria, archaea and without Phylum identification ASVs were removed from the ASV table using the phyloseq package [41]. For the metrics of α-diversity of bulk soil, rhizosphere soil and root endosphere we applied rarefaction to an even sampling depth of 3168 reads to all the samples, except for an endosphere sample from a plot of site H2 which had low number of reads. Richness (observed ASVs) and Shannon Diversity index were calculated using the function “plot_richness” in phyloseq and Faith phylogenetic diversity (PD) using Phylomeasures package. To test whether the ASV richness and diversity differed due to the type of bacterial community, we applied aligned rank transform for non-parametric factorial data with site as random factor using the ARTool package [42]. We applied this test because the data were not normally distributed and to consider that sample replicates are nested in the site. To test the effect of elevation, we applied the same analysis with the stage of succession and site as random effects. When the fixed effect was significant, a pairwise comparison of means was carried out using contrasts in emmeans package [43]. To analyze the effects of inundation on the three types of bacterial communities, we separated the endosphere samples from the rhizosphere soil and bulk soil samples. The endosphere samples were rarefied to 3168 sequences, whereas bulk soil and rhizosphere soil samples were rarified to 4520 sequence library depth to increase sequence sampling in more diverse samples. For all β-diversity analyses among different types of samples and elevations, the ASV tables were normalized calculating the relative abundances by dividing the raw abundances by the total number of counts per sample to prior to calculate the Bray–Curtis and UniFrac weighted and unweighted distance matrix using a vegan package [44,45]. The pattern of clustering of bacterial communities from bulk soil, rhizosphere soil and root endosphere was visualized in PCoA plots. Type of bacterial community, elevation and stage of succession effect on bacterial community composition was tested on all distance matrices by permutational analysis of variance (PERMANOVA) using the “Adonis” function, and dispersion of variance tests were performed with the function “betadisper” with the R vegan package [44].

#### 2.6.2. Environmental Variables and Bacterial Communities

To visualize which environmental variables and plant traits were influencing the bacterial community compositions, we constructed a Canonical Analysis of Principal coordinates (CAP) based on the Bray–Curtis dissimilarity distances with the ordination function in “phyloseq”. Then, we obtained a stepwise model from the constrained ordination method to know which environmental parameters were influencing the bacterial community composition with the function “ordistep” in the vegan package. A prior collinearity test of the variables was performed with the Hmisc package. Moreover, a mantel test was used to detect linear relationships between dissimilarity matrices of the plant traits and plant-associated bacteria in endosphere and rhizosphere with the function “mantel” with Pearson correlation method in the “vegan” package.

#### 2.6.3. Taxonomic Composition and Endosphere Analyses

Relative abundances of the major taxa were visualized in bar plots and differences of phyla sample types were calculated using a Kruskal–Wallis test with a false discovery rate (FDR) correction using the function “summarize_taxonomy” from the mctools package. To identify the phyla whose relative abundance were associated to either HE or LE elevation in each type of sample, we applied a stepwise algorithm implemented in the “selbal” package [46]. This method was selected for the advantages to preserve the compositional data principles and that the effect of the stage of succession can be considered as a covariate in the model. The abundance dataset of the ten more abundant phyla along with a group named “other” comprising the low abundant phyla were added to the model and the most optimal variables were represented graphically. Moreover, to asses which ASVs were responsible for the dissimilarity among elevations in the three community types we applied the similarity percentage (SIMPER) method based on Bray–Curtis dissimilarity distance [47] using the software PAST (version 3.25, [48]). This method ranked the ASVs which contributed to the dissimilarity among elevations and we selected the top 30 ASV and their abundance was scaled by site to be visualized in a heatmap with the function “heatmap.2” of the ggplot package.

Further we investigated the endosphere bacterial communities in more detail. First we calculated the proportion of taxa found exclusively in endosphere and the proportion of ASVs shared among the three types of samples using UpSet package [49] and visualized them in Venn diagrams. A similar approach was used to calculate and visualize the shared endosphere ASVs among elevations. In order to determine the endosphere taxa closely associated to *E. atherica* and disentangle how these taxa vary according to elevational ecotypes, we filtered the ASV endosphere dataset to obtain the endosphere core bacterial community. The filtering was based on ASVs present in 90% which accounted for at least 0.0001% of the total ASV relative abundance of the endosphere samples. The relative abundance of the core community in endosphere samples was compared among elevations using a *t*-test using a “t.test” function.

#### 2.6.4. Putative Functional Profile

Functional profiles of bacteria ASVs were predicted using the FAPROTAX (v1.2: [50]) database, from which nine functions relevant for this study were selected: cellulolysis, chitinolysis, fermentation, ligninolysis, nitrification, nitrogen fixation, ureolysis and xylanolysis. For this, the bulk soil and rhizosphere samples were normalized to 10,118 and 4520 sequences, respectively, to increase sampling depth in more diverse communities. Differences on the predicted functional profiles among elevations in each compartment were assessed by a Welch *t*-test using a “t.test” function in R. Potential denitrification activity was measured following [51] in collaboration with the University of Lyon.

## 3. Results

### 3.1. Bacterial Community Composition

After the archaeal, mitochondria and chloroplast sequences removal a total of 35,835 unique ASVs across 54 samples were obtained. Bacterial communities associated with the root endosphere showed the lowest values in alpha diversity (Richness F = 57.3, df = 2, *p* < 0.001; Shannon F = 56.4, df = 2, *p* < 0.001; PD F = 61.2, df = 2, *p* < 0.001), whereas rhizosphere and bulk soil did not differ from each other. Elevation did not affect ASV richness and diversity (Supplementary Material S5–S7). Only the phylogenetic composition of ASVs in soil was significantly higher in HE sites (F = 14.562, df = 1, *p* = 0.046).

**Figure 1:**
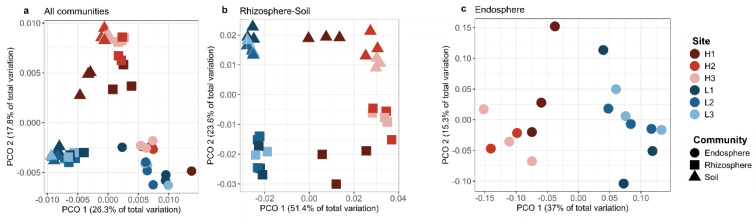
Principal coordinate analysis based on weighted Unifrac distances of the bacterial community inhabiting the endosphere (spheres), rhizosphere (squares) and bulk soil (triangles) (**a**). Types of communities were split in two plots to observe the effect of elevation differences on rhizosphere and soil (**b**) and on endosphere samples (**c**). Percentage of community variance explained by each axis is indicated in parentheses and PERMANOVA pseudo-F and *p*-values for elevation and compartment effect are reported in the text. Symbol color indicate sites, symbols in red shades are found at high elevation and blue shade at low elevation.

Bacterial community structure differed between bulk soil, rhizosphere and root endosphere (Unifrac weighted, Figure 1). The endosphere had a different community compared to the rhizosphere and soil at each elevation (Type of community, pseudo-F = 6.04, *p* < 0.001, Figure 1a). Elevation had a clear effect on the bacteria in soil and rhizosphere (Figure 1b; Elevation, df = 1, pseudo-F = 28.3, *p* < 0.001). The PCO1 axis explained 51.4% of the variability, while the PCO2 axis showed the dissimilarity in bacteria composition between type of community (Figure 1b; Type of community, df = 1, pseudo-F = 10.44, *p* < 0.001). Elevation affected clustering in the endosphere communities (Elevation df = 1, pseudo-F = 3.1, *p* = 0.027). Moreover, a difference between soil and rhizosphere bacterial communities due to stage of succession was observed (age of succession, df = 2, pseudo-F = 13.9, *p* < 0.001), but this difference was only between H1 (78 years old) and H2-H3 (53 years old), whereas no effect on stage of succession among LE sites was observed. Endosphere bacterial composition did not differ according to stage of succession. Similar clustering patterns were clear in the ordination of unweighted UniFrac and Bray–Curtis distances, suggesting that low abundance ASVs also contributed to differences among elevations (Supplementary Material S8).

### 3.2. Relationship between Environmental Variables and Bacterial Composition

**Figure 2:**
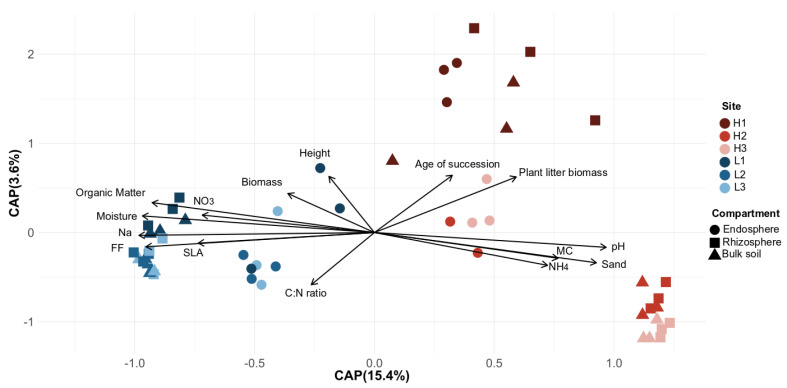
Partial distance-based redundancy analysis (db-RDA) for the bacterial communities associated with *E. atherica* traits and environmental variables based on the Bray–Curtis dissimilarity. SLA, specific leaf area; Biomass, aboveground biomass; MC, Mycorrhizal colonization. C:N ratio, soil total carbon to total nitrogen ratio. FF, Flooding frequency. Percentage of variance explained by each axis is indicated in parentheses. Symbol color indicate sites, symbols in red shades are found at high elevation and blue shade at low elevation.

Several site, plant and soil characteristics were driven by elevation, and soil and associated root bacterial communities followed the same pattern. HE sites had a higher percentage of sand, pH and ammonium content, which in turn resulted in a high mycorrhizal colonization and a strong influence on bacterial community structure (Figure 2, Supplementary Material S9). Bacterial communities in H1 clustered apart from H2 and H3 due to the stage of vegetation succession, higher plant litter mass and lower C:N ratio found in this site. LE sites had a higher organic matter, moisture, sodium and nitrate content. These soil characteristics together with flooding with salt water influenced plant responses to inundation (e.g., higher specific leaf area), as well as plant–bacteria associations. The effect of environmental variables on bacterial communities was greater at LE, given that soil, rhizosphere and endosphere bacterial structures clustered together (Figure 2). Stage of succession (F = 2.00, *p =* 0.04) and ammonium content (F = 1.59, *p* = 0.04) had a strongest influence on the soil bacterial community, whereas elevation (F = 2.54, *p* = 0.010) and ammonium content (F = 1.46, *p* = 0.025) strongly impacted the rhizosphere bacterial community. Moreover, rhizosphere but not endosphere bacterial communities were associated with plant traits (mantel_rhizo_ vs. _plant_-r = −0.288, *p* = 0.005; mantel_endo_ vs. _plant_-r = 0.091, *p* = 0.188).

### 3.3. Identification of Taxa with Differential Abundance among Elevations

**Figure 3:**
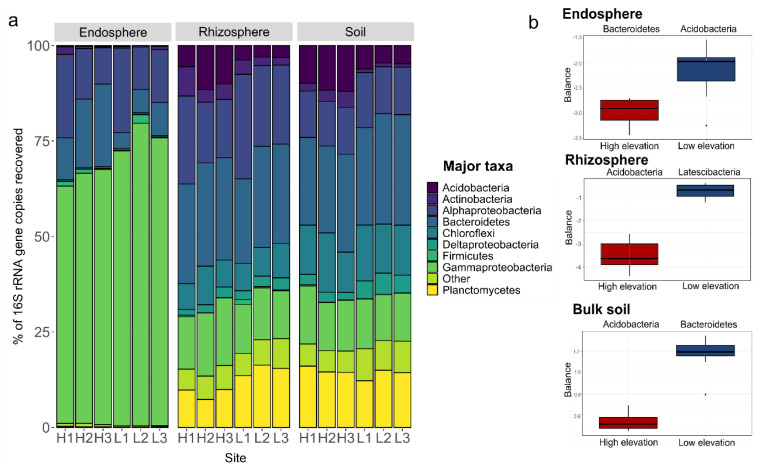
Relative ASV composition in *Elytrigia atherica* endosphere, rhizosphere and soil from each site at high salt marsh elevation (sites H1–H3) and low elevation (L1–L3). (**a**) Bar plot showing the phyla with at least 0.5% abundance, “Other” indicate the group of taxa with less than 0.5% abundance. (**b**) Phyla associated with either high or low elevation in each type of sample. Box plot shows root-mean-squared error resulted of the forward selection method. The apparent discrimination accuracy of these balances is 1.

Taxa abundance differed among the three type of communities (Figure 3). For instance, Proteobacteria abundance was significantly higher in the rhizosphere than in soil, 37.9% and 29.2%, respectively (KW FDR, *p* < 0.001), whereas Chloroflexi, Acidobacteria and Planctomyces were more abundant in soil (KW FDR, *p* < 0.001) than rhizosphere. The most dominant taxa in the endosphere was Proteobacteria and this phylum was more represented in LE sites compared to HE sites, comprising 91.3% of the ASV and 79.3%, respectively.

Balance analyses revealed that Acidobacteria were relevant for distinction of bacterial communities at each elevation in the three types of communities. In the endosphere this phylum was more associated to LE whereas in the rhizosphere and bulk soil it was more associated to HE.

**Figure 4:**
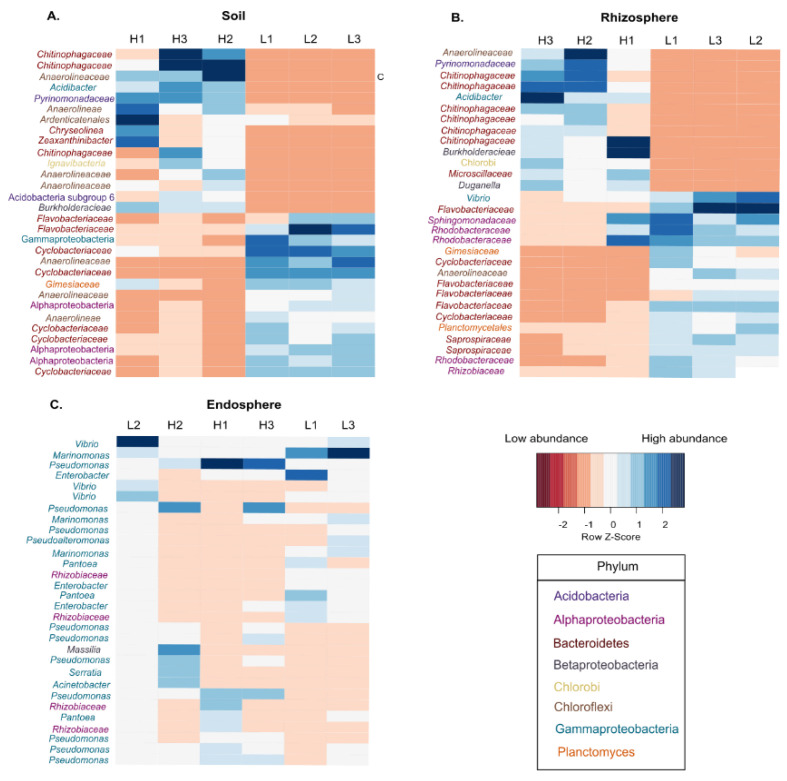
Heat-maps based on pair-wise SIMPER analysis showing the scaled abundances of the first 30 amplicon sequence variants (ASVs) that are primarily responsible for differences in bacterial community composition between high and low elevation in three types of communities: Soil (**A**), Rhizosphere (**B**) and endosphere (**C**). Sites label is located at the top of each column (H = high elevation; L = low elevation). The colors of the taxa names indicate the Phylum to which they belong.

In soil, the 30 ASVs ranked as the principal ASVs that explained the separation between elevations represented 10.21% of the total Bray–Curtis dissimilarity metric (Figure 4). In HE soils higher abundances were found of *Chitinophagaceae*, Acidobacteria, *Burkholderiaceae* and *Anaerolinaceae*, while in LE soils *Cyclobacteriaceae*, Alphaproteobacteria and *Flavobacteriaceae* were more abundant. In the rhizosphere, these 30 ASVs comprised 10.22% of the total variance. The predominant taxa found at HE were *Chitinophagaceae* and Betaproteobacteria, whereas in LE these were *Flavobacteriaceae*, *Saprospiraceae* and Alphaproteobaceria such as *Rhodobacteraceae*, *Sphingomonadaceae* and *Vibrio*. In the root endosphere, the selected ASVs comprised 65% of the total variance, with *Rhizobiaceae* being more abundant in HE, and genera considered as marine, such as *Vibrio* and *Marinomonas* in LE. However, in this type of community, the differences in composition were at lower taxonomic level because some genera were found at both elevations, for example, *Pseudomonas* and *Pantoea*.

### 3.4. Relationship Between External Sources in Each Plant Ecotype and Identification of Common Taxa in Endosphere

**Figure 5:**
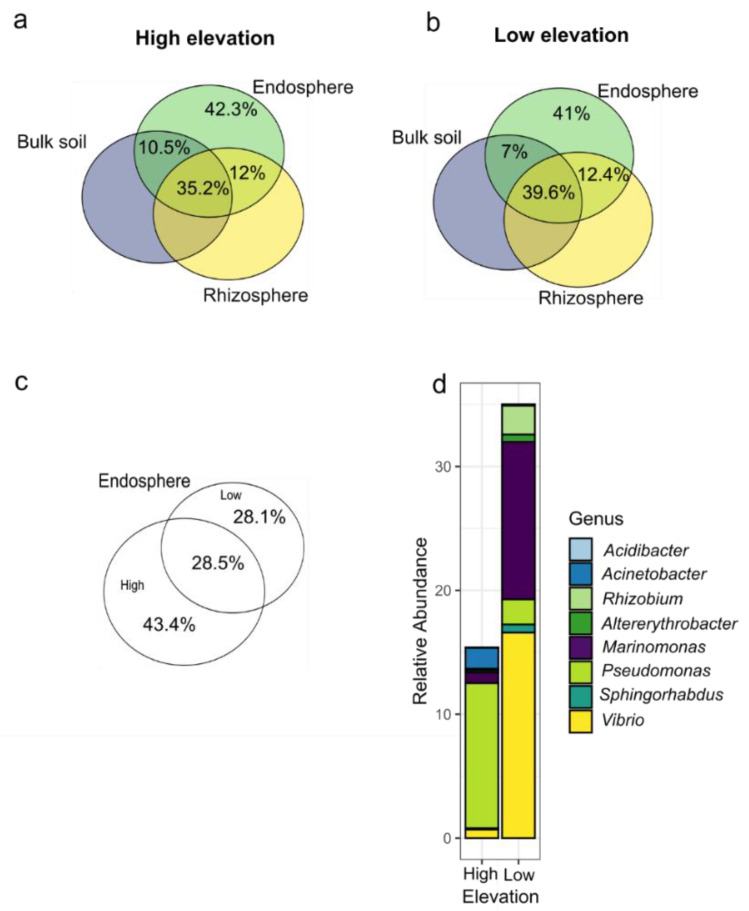
Differences in endosphere bacterial composition in each *E. atherica* ecotype. Venn diagram showing the proportion of the endosphere ASVs shared with rhizosphere and soil and exclusively found in inner root in HE ecotype (panel **a**) and LE ecotype (panel **b**). Venn diagram showing the proportion of shared endosphere ASVs among elevations (panel **c**). Mean relative abundance of the core bacterial genera at each elevation (panel **d**).

We observed that both HE and LE endospheres shared around 60% of the ASVs with bulk and rhizosphere soil, while the rest was exclusive to root internal tissue (Figure 5a,b). Moreover, ecotypes only shared 28.5% of ASVs in their root endosphere. The majority of non-shared ASVs were found in the endosphere community at HE (43.4%) compared to 28.1% in the endosphere at LE (Figure 5c). Importantly, the majority of ASVs that were exclusive to the roots were not found in the inner root of the other ecotype. For instance, in HE 85.5% of the total non-shared ASVs were found exclusively in HE and in LE was 75.6%. Together these results indicate that although endosphere communities at both elevations were shaped by the availability of local bacteria—as the majority of ASVs are transmitted horizontally from the exterior soil—those that are endosphere specific (around a third of their ASVs) are also ecotype specific.

In order to identify which bacteria taxa were associated to *E. atherica* regardless of their ecotype, we obtained the core endosphere. The core endosphere comprised 10 ASVs corresponding to 8 genera: *Acidibacter*, *Acinetobacter*, *Altererytrobacter, Marinomonas*, *Pseudomonas* (2 ASVs), *Rhizobium*, *Sphigorhabdus* and *Vibrio* (2 ASVs) which together represented 18.9% of total abundance in H1, 10.2% in H2, 17.1% in H3, 22.4% in L1, 49.4% in L2 and 33.2% in L3. The abundance of the core was higher in LE (35% ± 11) compared to HE (15.4% ± 3.73) (t = −2.76, *p* = 0.014) mainly due to enrichment of *Marinomonas* and *Vibrio* (Figure 5d).

### 3.5. Potential Functions in Soil, Rhizosphere and Endosphere

**Figure 6:**
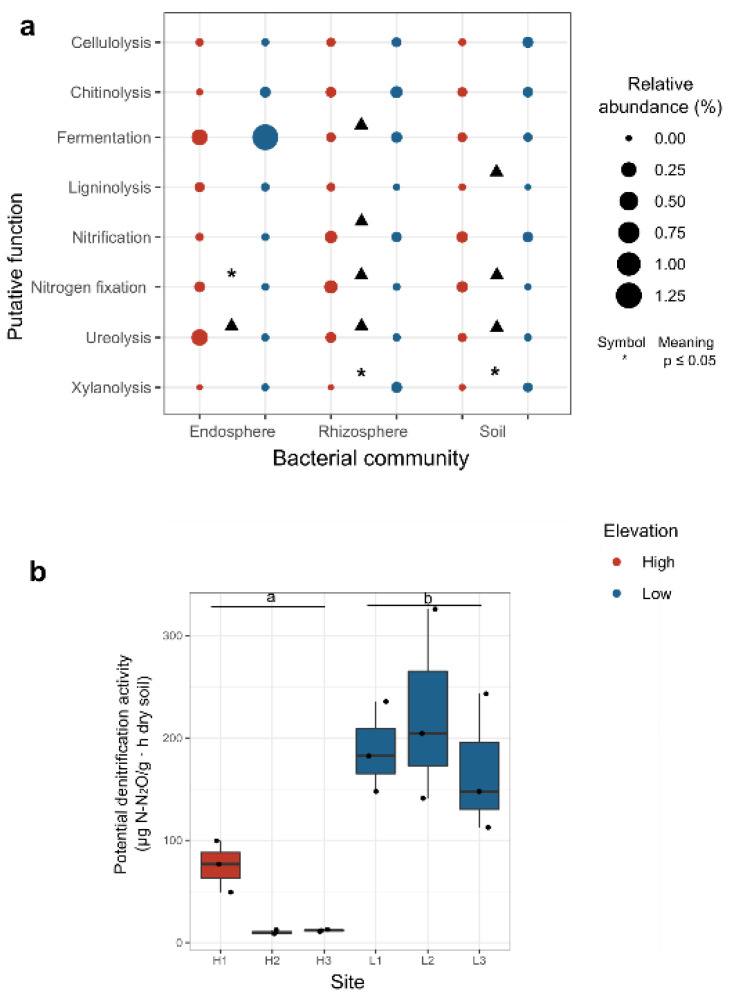
Potential function differences among elevations in each type of community. (**a**) Relative abundance of different functions based on the FAPROTAX database in the three types of communities. Elevation effect was tested with aligned rank transform for non-parametric factorial and significant differences are indicated with asterisks. Triangle symbols indicate potential functions that were increased in sites with a tendency to be higher in high or low elevation sites. (**b**) Potential soil denitrification activity. Different letters indicate significant difference among elevations (t-Welch test *p* < 0.05).

Functions related to organic matter degradation, such as rhizosphere xylanolysis, increased in LE. Soil ligninolysis tended to be higher in HE, while fermentation tended to be higher in LE (Figure 6a, Supplementary Material S10 and S11, which can be related to the higher organic matter content in LE and more plant litter in HE. Processes related to N-cycling, such as nitrogen fixation was enhanced in the HE endosphere, and also in the soil and rhizosphere but only in HE 53-year-old sites (Supplementary Material S11). A higher abundance of bacteria performing nitrification was found in the rhizophere in 53-year-old sites. Potential denitrification activity was higher in LE soils than HE soils (t.ratio = −3.17, df = 3.59, *p* = 0.039) (Figure 6b).

## 4. Discussion

Significant environmental changes pose challenges for plants, as they must respond through ecological and/or evolutionary adaptations. For instance, plant (genetic) adaptation to new environmental conditions might affect plant–microbiome interactions whereas the capacity of microorganisms to rapidly adapt environmental and host changes can alter host evolution [5]. Therefore, understanding the eco-evolutionary dynamics of plant–microbe interactions is crucial to predict the adaptation of plants to new stressful environmental condition. We studied the interaction of the native invasive grass *Elytrigia atherica,* at high and low salt marsh inundation frequency. Previously, *E. atherica* exclusively dominated at low inundation frequency at sites situated high above sea level, but for some decades now it has spread to low elevation sites, causing a decrease in the diversity of plants down the elevational gradient [21]. The differences in duration and frequency of tidal inundations across the salt marsh elevation gradient cause differences in soil physico-chemical conditions and consequently, affect plant–microbiome interactions.

### 4.1. Elevation Influences Soil Parameters, Which in Turn Strongly Modulate Soil Bacterial Communities

Multiple edaphic factors, such as pH, moisture and organic matter content shape soil bacterial community composition. In the soil of LE we observed higher contents of clay, sodium, moisture, organic matter and nitrates than in that of HE, which is in accordance with previous studies in Wadden sea salt marshes [19,52]. Despite the richer nutrient content observed in LE sites, this did not result in higher bacterial richness in bulk soils compared to HE soils, as indicated by lower bacterial phylogenetic diversity in LE compared to HE. Given that phyla such as Acidobacteria and Actinobacteria, which are less tolerant to sodium-rich and waterlogged conditions [53], showed an evident decline from HE to LE sites, we speculate that the lower diversity observed in LE is caused by the decrease in the abundance of bacterial phyla sensitive to sodium, which is high in LE soils [54].

In addition to the effects of soil edaphic factors on soil microbiome along salt marsh elevation, the high variability in flooding frequency—which translates into variation in soil aeration and salinity—influences soil parameters along vegetation successional stages [55,56]. Our results indeed showed that the bulk soil bacterial composition was correlated to successional stage, and we suggest that impact of plant litter quantity and quality might explain this relationship. Plant litter mass was higher in HE sites, with the oldest site (H1) having the highest amount of litter. This accumulation of litter through time is due to dominance of *E. atherica* as this species produces recalcitrant litter with a low decomposition rate [57,58], hampering carbon incorporation into the soil [58,59]. Moreover, plant litter accumulation is low at LE as it is partly washed away by the tides. In addition, presence of high-quality litter from subdominant plant species varies across successional stages and elevation, hence, their litter quality and root-exudates also may influence the dissimilarity of saprophytic microbial communities found in bulk soils [59,60]. Overall, the observed effects of elevation and successional gradient on soil bacterial communities are in agreement with previous findings that identified sodium and organic matter content as the most important abiotic factors driving the soil microbiome assembly within a site and across successional stages, respectively [56].

### 4.2. Plant–Microbial Interaction Changes with Flooding Frequency and Plant Phenotypes

Salt marsh elevation modulates the composition of bacterial communities associated with the rhizosphere both indirectly—i.e., via the impact of elevation on bulk soil communities that act as local bacterial species pool (see previous section)—and directly, through differences in exudation patterns and litter quality which are often associated with different plant species. In this study we demonstrated that plant ecotypes also have an important, direct impact on host-microbial interactions and rhizosphere community composition, given that each *E. atherica* ecotype, i.e., growing at HE or LE, harbors a distinct rhizosphere bacterial community. In the case of the LE ecotype, differences in rhizosphere composition could be explained by the ability of this ecotype to grow fast in response to higher rich-nutrient soil [61], as indicated by its higher SLA. Regarding the HE ecotype, the differentiation in rhizosphere community composition was explained by the difference in the endomycorrhizal colonization, which its known to contribute to the rhizosphere bacterial community assemblage by stimulating some species while suppressing others [62,63]. Studies focusing on the bacterial community composition associated with the rhizosphere of *Spartina alterniflora,* another dominant and invasive grass species in salt marshes [64,65], indicated that genotypically distinct growth forms harbor different bacterial communities [66], corroborating our evidence on the potential role plant phenotypes in microbiome selection in the rhizosphere. This microbiome differentiation as a function of genotype and phenotype in both *S. alterniflora* and *E. atherica* [21,58,67,68] leads us to suggest that the plant microbiome may play an important role in allowing these invasive species to successfully spread across the salt marsh.

### 4.3. Core Endosphere Composition and Proportion of Bacteria Exclusive to Each Ecotype

Comparisons between rhizosphere and endosphere microbial communities have revealed that as the interactions between plants and microbes intensifies, i.e., when microbes can actively colonize the inner part of the plants as endophytes, the plant imposes a strong selection pressure that limits microbiome composition [69]. Our results corroborate these findings given the reduction in richness and diversity in the bacterial communities colonizing the root endosphere in relation to corresponding rhizosphere community, likely due to the selection for bacterial traits that allow them to enter and persist inside the roots [69,70,71]. Our data also revealed that a large percentage of the endophytes were likely transmitted horizontally, i.e., they have been acquired from the surrounding soil, as the majority of the root endosphere (60% of the ASVs) was shared with either rhizosphere or bulk soil communities [71,72]. The majority of the reminder endosphere ASVs were specific to each ecotype, indicating the indirect effect of elevation on plant physiology influencing the endosphere composition. Although we cannot pinpoint the exact plant mechanisms leading to the differentiation in endosphere composition observed among plant ecotypes, several aspects might be at play. For instance, the difference in composition may be explained by differences in the endosymbiotic bacteria associated to the mycorrhiza fungus [62], which are lacking or not detected in the LE ecotype. Furthermore, this indicates the strong effect of elevation on plant local adaptation since plant genetic or phenotypic relatedness is positive correlated to root bacteria similarity [66,73]. Although ecotype-specific bacteria could contribute to the better performance of seedlings in the elevation that resembled their parental origin [23], this hypothesis needs to be specifically tested in further studies.

### 4.4. Differences in Microbial Functional Composition Revealed Potential Role in Plant Stress Tolerance

A prerequisite for accepting the hypothesis that the bacterial communities associated with the endosphere of each plant ecotype are linked to their adaptation to environmental conditions consists in the ability of plants to select for bacterial traits that alleviate stress. For instance, nitrogen is often the limiting soil nutrients restraining plant growth in saline environments [74], as plants also use nitrogen to increase salt tolerance by synthetizing nitrogen compounds [14]. In this context, interaction with microbes could contribute to nitrogen acquisition, as demonstrated by the higher abundance of taxa with the potential ability to fix atmospheric nitrogen in the endosphere of ecotypes from HE, which showed lower soil nitrogen content, especially in 53-year-old sites [75].

The most abundant taxa found in the root endosphere, i.e., Gammaprotobacteria, Alphaproteobacteria and Bacterioidetes, are known as successful root colonizers in multiple plant species [11,76]. Many of these endophytic genera positively impact plant growth by e.g., providing phytohormones, such as indole acetic acid, or modulating production of reactive oxygen species [69,73,77]. Notably, we observed an enrichment of marine genera such as *Marinomonas* sp. and *Vibrio* sp.—bacterial species that are associated with the endosphere of halophytes—in ecotypes from LE [34,78]. There is evidence that *Vibrio* sp. can contribute to plant growth [78,79] by synthesizing 1-Aminocyclopropane-1-carboxylate (ACC) deaminase, which reduces the accumulation of ethylene in the plant, thus alleviating a common stress response (growth inhibition) associated with salinity [80]. Therefore, a higher abundance of *Vibrio* in LE ecotype may indicate a higher ability of this ecotype to alleviate saline stress, which agrees with higher sodium content at LE elevation.

Finally, the difference in bacterial-ecotype interaction was also mirrored to other microbes, as observed for the endomycorrhiza-ecotype interaction. The association of mycorrhiza with *E. atherica* is facultative [81]; hence, in waterlogged, very dry or saline soils colonization it is not favored due to low mycorrhizal inoculum potential [82,83], explaining the low colonization in LE plants. However, even in low mycorrhizal colonization, mycorrhiza might increase *E. atherica* tolerance to higher soil sodium content in LE [84]. Moreover, high marsh sites can be dry during summer, and a high level of mycorrhizal colonization may improve water uptake in HE ecotype, such as in other salt marsh species [85].

## 5. Conclusions

We showed that ecotypes of *E. atherica* differ in root endosphere and rhizosphere bacteria composition. This difference in bacterial community composition as a function of saltwater inundation frequency is partly due to direct effects of flooding frequency on plant phenotype, as well as the indirect effect of salinity differences on soil bacterial communities. The LE ecotype harbors more halophylic bacterial species that may contribute to the saline stress alleviation, while the HE ecotype may be more associated to endomycorrhiza species to increase water uptake in dry summer periods. Together the results suggest that the dynamic genotypic and phenotypic adaptation of this holobiont to diverse local salt marsh conditions can be also linked with the establishment of microbial interactions with local free-living microorganisms.

## Figures and Tables

**Table 1:** Location and characteristics of the sample sites at the salt marsh of Schiermonnikoog, the Netherlands. Elevation is showed in Amsterdam Ordnance Data (mAOD) units. Chronosequence age is expressed in years after establishment. Flooding frequency is expressed as the annual proportion of inundated time. At each site the dominant and subdominant vegetation was recorded.

Site	Plot	Latitude	Longitude	Stage of Succession (Years)	Absolute Elevation m (AOD)	Flooding Frequency	Plant Species	
							Dominant	Subdominant
H1	A	53.4889	6.22336	78	1.722	0.019	*E. atherica*	*Atriplex prostata*
	B	53.489	6.22334	78	1.694	0.022		
	C	53.4889	6.22328	78	1.693	0.022		
H2	A	53.4944	6.26251	53	1.704	0.021	*E. atherica*	*Festuca rubra, Artemisa maritima*
	B	53.4944	6.26239	53	1.71	0.02		
	C	53.4945	6.26234	53	1.708	0.02		
H3	A	53.4947	6.27328	53	1.806	0.014	*E. atherica*	*F. rubra, A. maritima*
	B	53.4948	6.27344	53	1.887	0.011		
	C	53.4948	6.27332	53	1.959	0.009		
L1	A	53.4793	6.23653	78	1.373	0.072	*E. atherica*	*Atriplex portulacoides*
	B	53.4793	6.23655	78	1.404	0.064		
	C	53.4792	6.23652	78	1.349	0.078		
L2	A	53.4848	6.269	31	1.394	0.067	*E. atherica*	*A. maritima. Limonium vulgare*
	B	53.4848	6.26901	31	1.392	0.067		
	C	53.4849	6.26917	31	1.352	0.078		
L3	A	53.4884	6.27361	31	1.285	0.099	*E. atherica*	*A. marítima, A. portulacoides, L. vulgare*
	B	53.4883	6.27378	31	1.367	0.072		
	C	53.4884	6.2737	31	1.358	0.075		

## Supplementary Materials

The following Supplementary Materials are available online at https://www.mdpi.com/2076-2607/8/10/1619/s1, S1. Map showing the geographic location of the sampling sites in the barrier island of Schiermonnikoog, The Netherlands. S2. Data of the plant traits, intensity of mycorrhizal colonization and plant litter mass from each of the sampling sites. S3. Details of the soil physicochemical methodology. S4. Table showing the values of soil physicochemical parameters. S5. Boxplots displaying the richness of the Bacterial Amplicon Sequence Variants (ASVs) comparing communities (panel A) and elevations (panel B-D). S6. Boxplots displaying the Shannon diversity index of the Bacterial Amplicon Sequence Variants (ASVs) comparing communities (panel A) and elevations (panel B-D). S7. Boxplots displaying the Faith phylogenetic diversity index of the Bacterial Amplicon Sequence Variants (ASVs) comparing communities (panel A) and elevations (panel B–D). S8. Principal coordinate analysis based on unweighted Unifrac and Bray-Curtis dissimilarity distances of the composition of bacterial communities from the root endosphere, rhizosphere and bulk soil (upper panel). In the lower panel, the table is showing the one-way permutational multivariate analysis of variance with the influence of elevation and community type on the variation in bacterial composition S9. Principal component analysis (PCA) showing the variation among sites in terms of soil physicochemical parameters and plant traits and environmental factors. S10. Potential functional differences among elevations in each type of community. S11. Barplots showing the relative abundance of the potential bacterial functions that showed a tendency to be higher in high or low elevation sites.
