# Supplementary material for: Salt Marsh Elevation Drives Root Microbial Composition of the Native Invasive Grass Elytrigia atherica
Source: Microorganisms. 2020 Oct 21;8(10):1619. doi: 10.3390/microorganisms8101619 (PMC7589393; doi:10.3390/microorganisms8101619)
Supplement: Supplementary file 1 [file microorganisms-08-01619-s001.pdf]

Supplementary material

Salt Marsh Elevation Drives Root Microbial Composition of the Native Invasive Grass *Elytrigia atherica*

Edisa García Hernández [1,*], Elena Baraza [2], Christian Smit [3], Matty P. Berg [3,4] and Joana Falcão Salles [1,*]

[1] Microbial Community Ecology, Groningen Institute for Evolutionary Life Sciences, University of Groningen, 9747 AG Groningen, The Netherlands

[2] Departamento de Biologia, Universitat de les Illes Balears-INAGEA, 07122 Mallorca, Spain; elena.baraza@uib.es

[3] Community and Conservation Ecology Group, Groningen Institute for Evolutionary Life Sciences, University of Groningen, 9747 AG Groningen, The Netherlands; c.smit@rug.nl (C.S.); m.p.berg@vu.nl (M.P.B.)

[4] Department of Ecological Science, Section Animal Ecology, Vrije Universiteit Amsterdam, 1081 HV Amsterdam, The Netherlands

* Correspondence: d.e.garcia-hernandez@rug.nl (E.G.H.); j.falcao.salles@rug.nl (J.F.S.); Tel.: +31-50-3632236 (E.G.H.); +31-50-36-32162 (J.F.S.)

S1. Map showing the geographic location of the sampling sites in the Dutch barrier island of Schiermonnikoog. Sites at High elevation are H1-3 and sites at low elevation L1-L3.

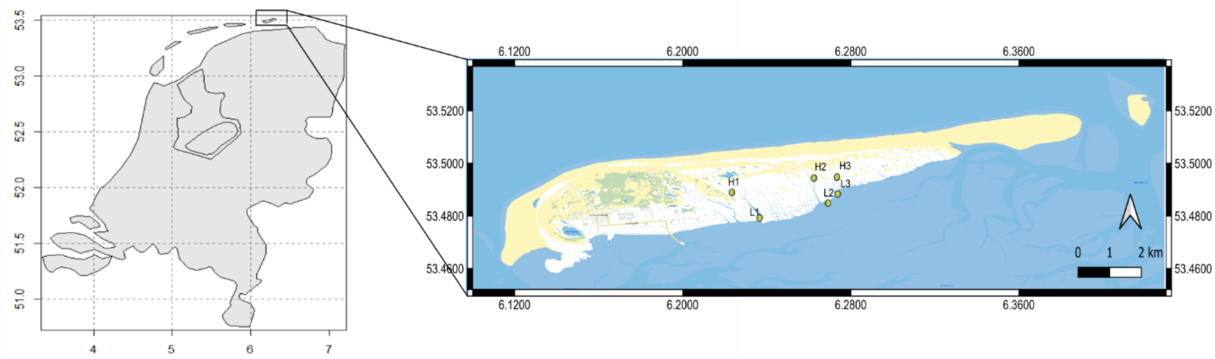

S2. Data of the plant traits, intensity of mycorrhizal colonization and plant litter biomass from each of the sampling sites. Sites H1-H3 are located at salt marsh high elevation and L1-L3 at low elevation.

**Site**	**Reproductive height (cm)**	**Specific Leaf Area (cm^2 of leaves g^{-1} dry mass)**	**Aboveground biomass (g)**	**Plant litter dry biomass**	**Intensity of mycorrhizal colonization (%)**
H1_A	85.35	100.6	39.35	50.71	9.33
H1_B	89.50	92.88	39.61	25.16	14.44
H1_C	89.10	97.55	29.4	43.94	33.33
L1_A	83.15	115.5	41.8	0.65	10
L1_B	83.15	142.34	49.59	0.68	5
L1_C	76.00	140.3	30.94	2.94	20.93
H2_A	70.55	85.34	17.18	17.85	27
H2_B	73.70	102.03	26.02	17.48	69.33
H2_C	72.05	98.24	10.07	15.91	20
L2_A	81.30	115.3	41.95	5.78	10
L2_B	90.80	100.63	40.1	9.05	4
L2_C	79.00	122.84	15.55	3.26	12
H3_A	74.70	109.05	37.87	19.17	38
H3_B	77.95	95.59	21.48	12.5	66
H3_C	82.55	96.95	25.51	12.69	42.22
L3_A	77.62	117.81	20.67	2.2	5.45
L3_B	71.75	121.41	31.79	4.08	0
L3_C	81.30	113.51	32.86	2.74	4

S3. Soil physicochemical parameters methods

The soil physicochemical parameters tested were texture, pH, soil water content (SWC), organic matter (OM), sodium (Na), nitrates (N-NO_3^-) and ammonium (N-NH_4^+), total carbon and nitrogen (TC/TN). The soil parameters were carried out in collaboration with the Department of Community and Conservation Ecology in the University of Groningen, except for texture that was performed in the Netherlands Institute of Ecology (NIOO-KNAW). Soil texture determined the grain size distribution by laser diffraction on a particle sizer (Malvern, Worchester, UK). The pH was measured weighting 15 g and adding 20 ml of distilled water. The tubes were shaken and left stand overnight, then the pH was measured using a potentiometer. Soil moisture was measured by oven-drying 10 g of soil at 105°C for ~16 h. Moisture percentage was calculated as fresh weight minus dry weight, divided by fresh weight multiplied by 100. After that, the dried samples were placed in a muffle furnace (Naberthermn, Germany) at 550 °C for 4 h. The soil organic matter content was calculated as dry soil weight – dry weigh after ignition, divided by dry soil weight x 100 [1]. To measure N content in nitrate and ammonium, 12.5 g soil was mixed with 30 ml KCl (1M), shaken for ~16 h using a custom-made overhead shaker (1 turn/s). Afterwards, the suspension was filtered with a paper filter by gravity and the extract was analyzed for N-NO^{3-} and N-NH^{4+} on a continuous flow auto analyzer (Type 5100; Skalar-40 BV, Breda, the Netherlands) using a colorimetric method [2]. For TC, TN and Na content, 10 g soil was first dried at 40 °C in a stove for 16 h and then ground to a fine powder in a Cyclotec 1093 mill. Sodium exchangeable ion content was measured by extraction of 5 g soil with ammonium acetate (1M, pH 7), mixed in the overhead shaker for 1 h and then filtered with a paper filter by gravity. The filtrate was analyzed on an atomic absorption spectrometer (AAS) (Varian Spectra AA 220FS, Australia). For TN and TC measurements, the soils were analyzed on a combustion elemental analyzer (CE Instruments EA 1110).

S4. Soil physicochemical parameters in each sampling site. TC; Total Soil Carbon percent, TN; total Nitrogen percent.

Site	Soil Organic Matter (%)	Soil water content (%)	Soil nitrates (mg·N-NO_3^-·g^{-1} dry soil)	Soil Ammonium (mg·N-NH_4^+·g^{-1} dry soil)	pH	Sodium (mg Na^+/100 g dry soil)	Sand (%)	TC (%)	TN (%)	Soil carbon/ nitrogen ratio
H1_A	11.71	28.56	23.15	5.44	8.05	249.41	29.33	4.809	0.408	11.8
H1_B	12.19	27.94	22.07	7.56	8.025	185.7	37.94	4.772	0.4	11.9
H1_C	10.94	28.69	13.31	6.41	8.26	194.9	35.17	4.937	0.43	11.5
L1_A	16	41.29	15.48	4.73	7.545	731.52	12.42	5.957	0.502	11.9
L1_B	16.39	41.47	37.85	1.86	7.61	753.9	13.69	5.77	0.485	11.9
L1_C	16.03	43.34	14.08	4.64	7.58	764.23	13.41	6.175	0.5	12.4
H2_A	4.16	14.58	3.68	10.21	8.81	39.75	84.43	5.323	0.457	11.7
H2_B	3.87	12.45	3.15	7.62	8.84	32.6	82.16	1.593	0.119	13.4
H2_C	3.96	12.54	5.57	9.03	8.72	30.26	78.64	1.639	0.131	12.5
L2_A	14.82	43.95	20.93	3.48	7.53	769.94	15.38	5.243	0.396	13.3
L2_B	13.5	40.97	40.41	7.49	7.58	656.58	22.35	4.788	0.373	12.9
L2_C	14.99	43.66	58.32	6.12	7.51	793.22	15.39	5.465	0.426	12.8
H3_A	2.96	9.92	3.21	10.89	8.42	15.21	87.88	1.424	0.114	12.5
H3_B	4.29	11.76	5.11	10.75	8.77	34.87	80.69	2.074	0.16	13
H3_C	3.75	10.35	3.77	10.73	8.65	22.24	83.86	1.287	0.097	13.2
L3_A	16.25	47.74	19.99	7.74	7.43	925.47	12.65	5.662	0.456	12.4
L3_B	14.6	41.52	23.47	6.6	7.59	738.22	14.45	5.107	0.382	13.4
L3_C	14.08	39.6	22.88	5.95	7.56	636.3	13.83	5.208	0.378	13.8

S5. Bacterial Amplicon Sequence Variants (ASVs) richness comparing type of communities (A) and in each community separately: bulk soil (B), rhizosphere (C) and endosphere (D) in each sampling site. Sites H1-H3 are located at salt marsh high elevation and L1-L3 at low elevation. Letters denotes significant differences after a pairwise comparison of the least square means (p<0.001, Tukey adjustment).

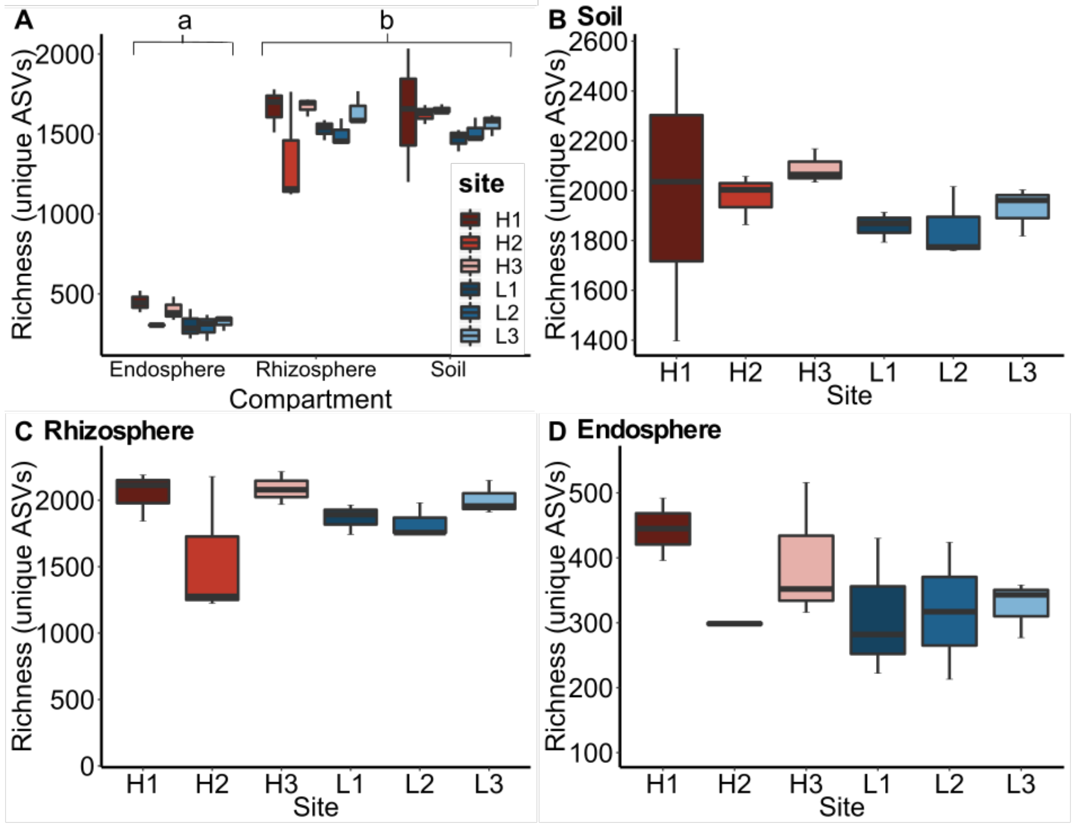

S6. Shannon diversity index of the Amplicon Sequence Variants (ASVs) comparing communities (A) and in each community separately: bulk soil (B), rhizosphere (C) and endosphere (D) in each sampling site. Sites H1-H3 are located at salt marsh high elevation and L1-L3 at low elevation. Letters denotes significant differences after a pairwise comparison of the least square means (*p*<0.001, Tukey adjustment).

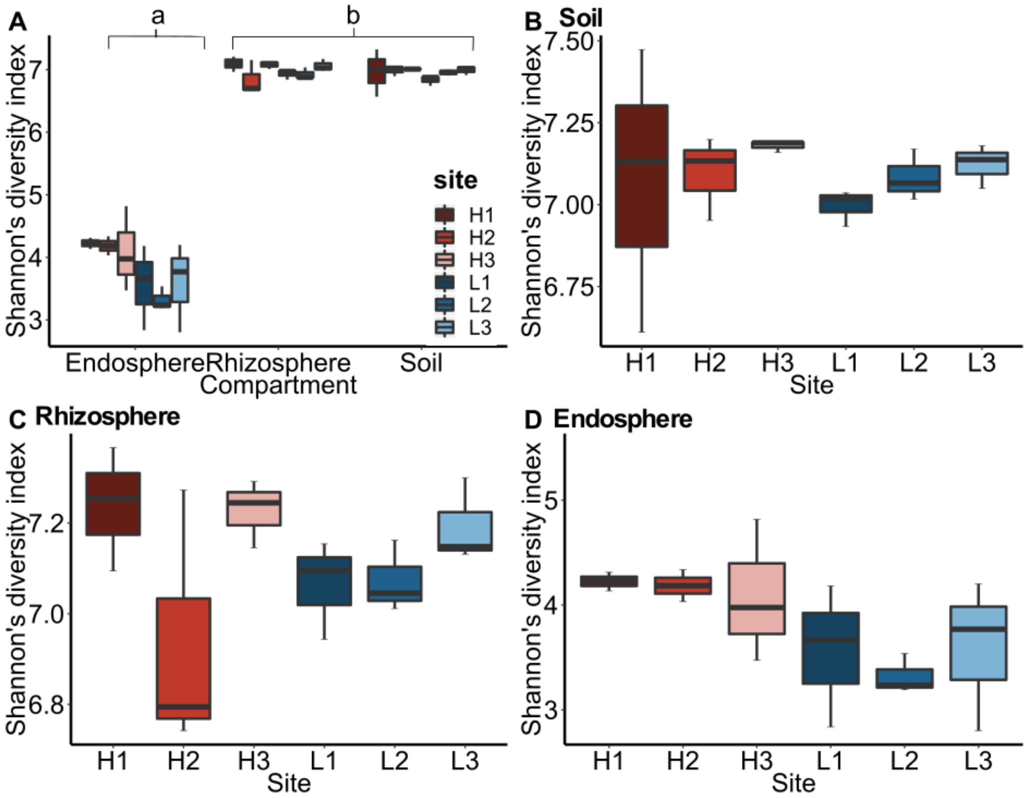

S7. Phylogenetic diversity index of the Amplicon Sequence Variants (ASVs) comparing communities (A) and in each community separately: bulk soil (B), rhizosphere (C) and endosphere (D) in each sampling site. Sites H1-H3 are located at salt marsh high elevation and L1-L3 at low elevation. In panel A and B, letters denote significant differences after a pairwise comparison of the least square means (*p*<0.001, Tukey adjustment), degrees of freedom method Kenward-Roger.

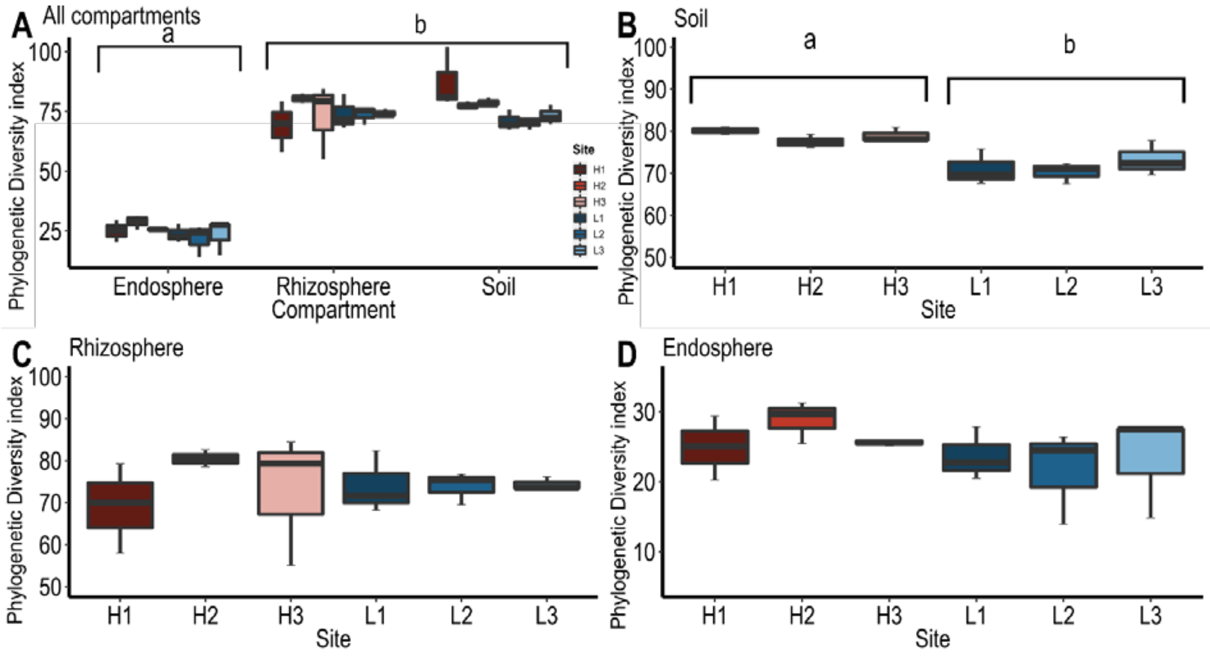

S8. Effect of elevation on soil, rhizosphere and endosphere bacterial community composition. Principal coordinate analysis based on unweighted Unifrac and Bray-Curtis dissimilarity distances of the bacterial community inhabit all type of communities i.e. endosphere, rhizosphere and soil as indicated at the top of each plot. Percentage of community variance explained by each axis is indicated in parentheses and summary of the permutational multivariate analysis of variance (PERMANOVA, 999 permutations) testing the effect of elevation, type of sample and stage of succession are reported in the table below the plots.

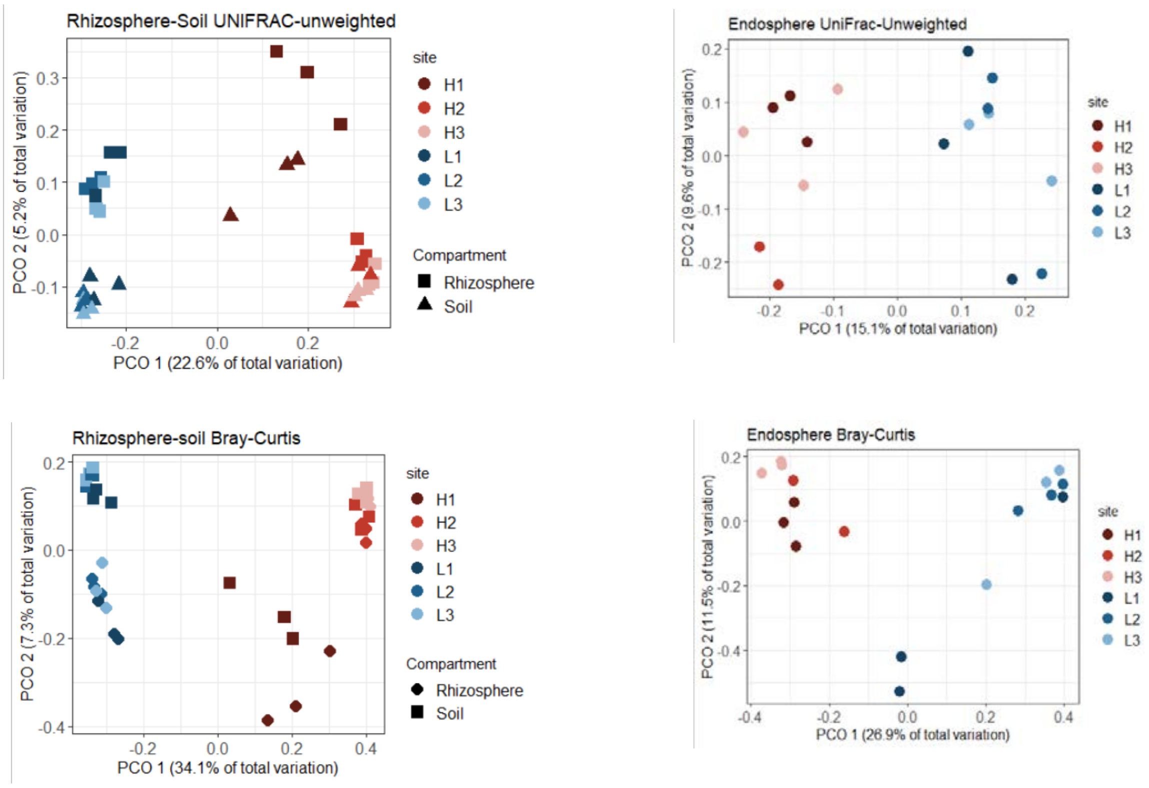

Type of community	Distance matrix	Effect tested	strata	Pseudo-F	Df	R^2	Significance	Significance dispersion of data
							(*p* value)	(*p* value)
Soil and rhizosphere	UniFrac unweighted	Elevation	Community	12.41	1	0.267	0.001	0.002
		Community	site	1.78	1	0.049	0.001	0.224
		Stage of succession	Community:Elevation	6.24	2	0.275	0.001	0.001
Endosphere		Elevation	-	2.28	1	0.132	0.001	0.411
		Stage of succession	Elevation	1.55	2	0.181	0.035	0.003
Soil and rhizosphere	Bray Curtis	Elevation	Community	16.26	1	0.323	0.001	0.001
		Community	site	2.03	1	0.056	0.001	0.098
		Stage of succession	Community:Elevation	8.35	2	0.336	0.001	0.003
Endosphere		Elevation	-	4.76	1	0.241	0.001	0.383
		Stage of succession	Elevation	2.82	2	0.287	0.017	0.314

S9. Principal component analysis (PCA) showing the variation among sites in terms of soil physicochemical parameters (A) and plant traits and environmental factors (B). Symbol color indicate sites, symbols in red shades are found at high elevation and blue shade at low elevation. Symbol shape depicts the age of successional stage. Arrows and their lengths indicate direction and strength of the environmental variables.

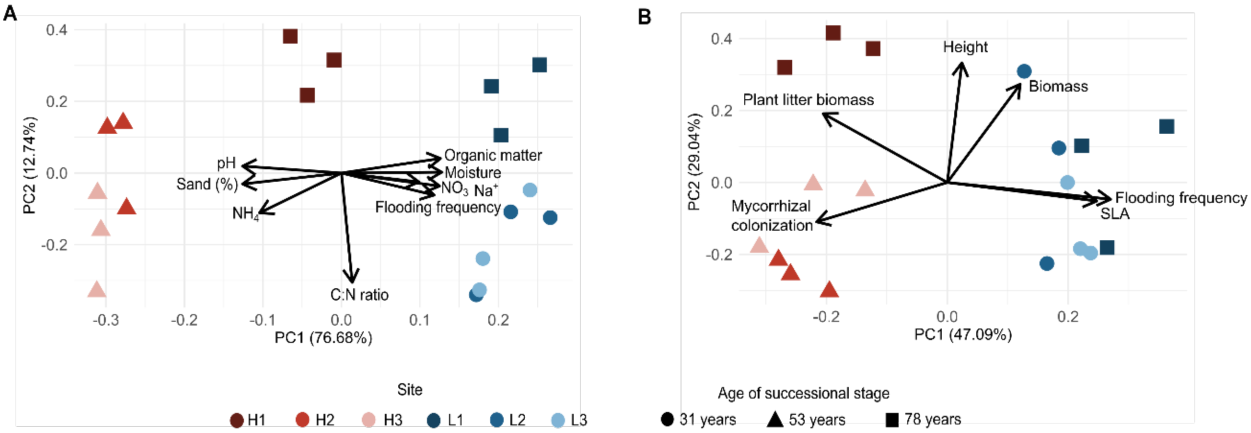

S10. Potential functional differences among elevations in each type of community. Summary of the aligned rank transform for non-parametric factorial with stage of succession as random factor analysis is showed. Values of $p<0.05$ were considered significant.

Community	Potential function	F	df	p-value		Higher abundance in:
Endosphere	Cellulolysis	0.02	1	0.908	NS	
Rhizosphere		6.61	1	0.103	NS	
Soil		7.16	1	0.058	NS	
Endosphere	Chitinolysis	0.94	1	0.421	NS	
Rhizosphere		0.54	1	0.529	NS	
Soil		0.17	1	0.717	NS	
Endosphere	Fermentation	5.79	1	0.117	NS	
Rhizosphere		5.79	1	0.117	NS	
Soil		0.16	1	0.721	NS	
Endosphere	Nitrification	0.02	1	0.905	NS	
Rhizosphere		7.16	1	0.058	NS	
Soil		6.10	1	0.119	NS	
Endosphere	Nitrogen fixation	8.06	1	0.048	*	High
Rhizosphere		7.16	1	0.058	NS	
Soil		5.79	1	0.117	NS	
Endosphere	Ureolysis	5.79	1	0.117	NS	
Rhizosphere		7.16	1	0.058	NS	
Soil		5.79	1	0.117	NS	
Endosphere	Xylanolysis	t=-1	8	0.347	NS	
Rhizosphere		15.54	1	0.018	*	Low
Soil		0.73	1	0.469	NS	
Endosphere	Ligninolysis	0.00	1	1.000	NS	
Rhizosphere		1.98	1	0.279	NS	
Soil		1.58	1	0.317	NS	

S11. Barplots showing relative abundance of the potential bacterial functions with a tendency to be higher in high or low elevation sites.

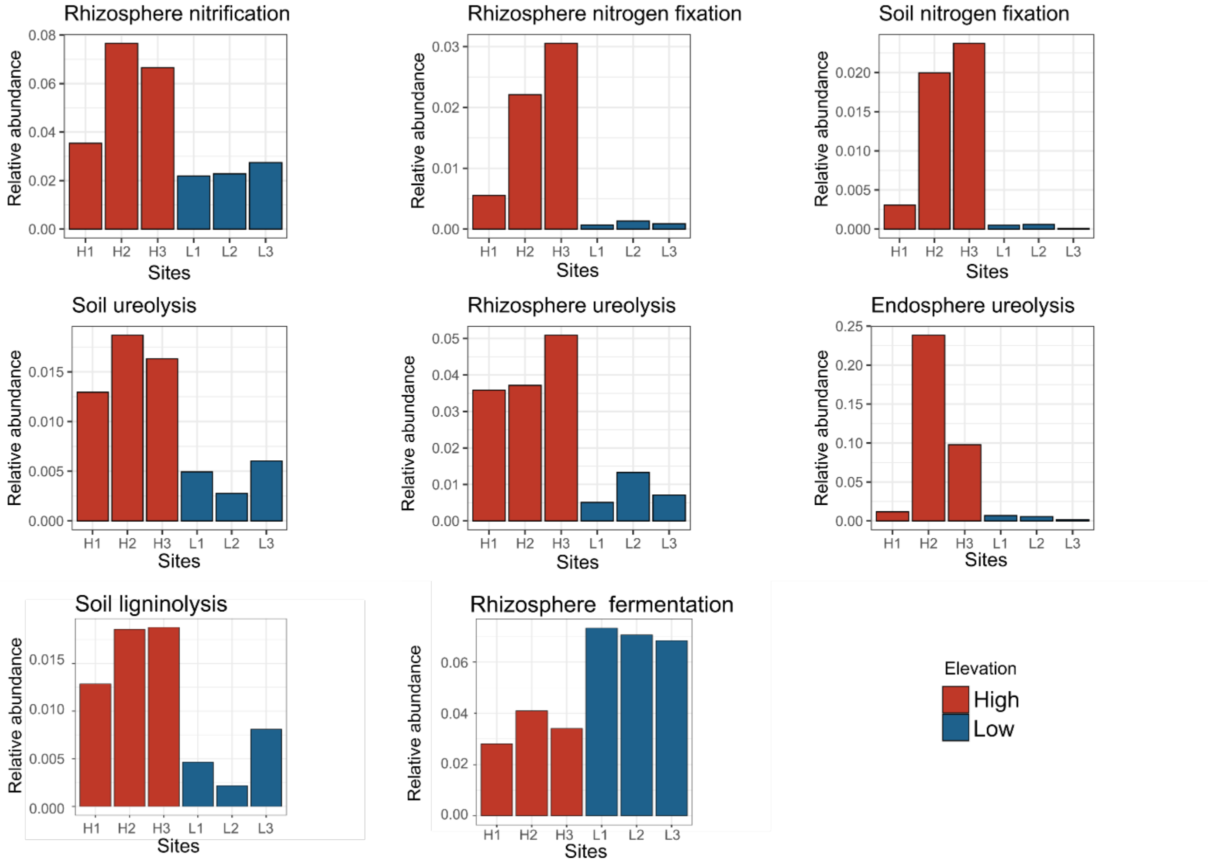

Literature cited

[1] B. Schulte, B.G. Hopkins, Estimation of soil organic matter by weight-loss-on-ignition, in: F.R. Magdoff (Ed.), Soil Org. Matter Anal. Interpret., SSSA Spec. Publ., WI, USA, 1996: pp. 349–359.

[2] D.R. Keeny, D.W. Nelson, Nitrogen - inorganic forms, in: A.L. Page, R.H. Miller, D.R. Keeny (Eds.), Methods Soil Anal., ASA-SSSA, Madison, Wisconsin, USA, 1982: pp. 643–698.
